# Implementation of an interruptive electronic health record alert improves optimal antibiotic prescribing in ambulatory patients with acute bacterial sinusitis: a quasi-experiment

**DOI:** 10.1017/ash.2025.10283

**Published:** 2026-01-19

**Authors:** Stormmy R. Boettcher, Anita B. Shallal, Adrienne Vaught, Steven T. Fried, John R. Craig, Brian M. Church, Rachel M. Kenney, Susan L. Davis, Michael P. Veve

**Affiliations:** 1 Department of Pharmacy, Henry Ford Hospital, Detroit, MI, USA; 2 Department of Pharmacy Practice, Eugene Applebaum College of Pharmacy and Health Sciences, Wayne State University, Detroit, MI, USA; 3 Division of Infectious Diseases, Henry Ford Hospital, Detroit, MI, USA; 4 Epic Pharmacy Helios Team, Henry Ford Health, Detroit, MI, USA; 5 Department of Internal Medicine, Henry Ford Hospital, Detroit, MI, USA; 6 Department of Otolaryngology- Head and Neck Surgery, Henry Ford Health, Detroit, MI, USA

## Abstract

Sinusitis is a leading cause for outpatient antibiotics. An interruptive electronic health record alert was implemented to promote optimal antibiotic selection and duration for acute bacterial sinusitis when suboptimal treatment is ordered. After implementation, optimal antibiotic prescribing significantly increased 7% preintervention versus 30% postintervention, (unadjOR, 5.69; 95% CI, 2.36 – 13.72; *P* < .001).

## Background

An estimated 775,000 antibiotic prescriptions are written for acute sinusitis each year, making it one of the leading reasons for antibiotic use in the United States.^1,2^ While most acute sinusitis cases reflect either viral etiology or noninfectious conditions, such as chronic rhinitis or migraines, antibiotics are still prescribed in greater than 80% of encounters.^1,3^ Of these antibiotic prescriptions, the majority are suboptimal when considering antibiotic selection and duration of therapy.^1^

The Centers of Disease Control and Prevention (CDC) Core Elements for Antibiotic Stewardship suggest identifying high-priority conditions where clinicians deviate from best-practices in antibiotic prescribing as targets for antimicrobial stewardship program (ASP) intervention.^4^ The Henry Ford Health (HFH) ambulatory ASP’s internal prescribing data suggested that acute sinusitis is a top five indication for an antibiotic prescription and that most are suboptimal regarding drug selection and duration of therapy. In response, the HFH ambulatory ASP developed an interruptive electronic health record (EHR) alert designed to improve drug selection and duration of therapy for outpatients with an acute bacterial sinusitis diagnosis. The study purpose was to describe the effect of a targeted sinusitis interruptive alert on optimal antibiotic prescribing at a large, urban health system.

## Methods

### Study design

This was a single pre, posttest quasi-experimental study conducted at HFH, a five-hospital health system with greater than 250 outpatient clinics across metropolitan Detroit, MI, USA. This study received institutional review board approval with a waiver of consent. Patients were included if they were greater than 18 years old, were treated in an ambulatory clinic setting for acute bacterial sinusitis, and received an antibiotic prescription for acute bacterial sinusitis during the study time frame. The preintervention period was from April 3, 2024, to August 1, 2024, and the corresponding postintervention period was from April 3, 2025, to August 1, 2025.

### Intervention

**Figure 1.**
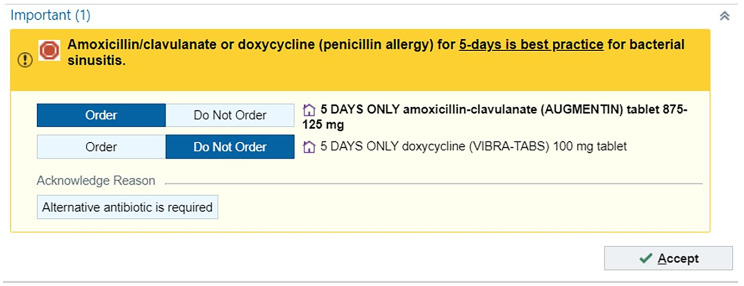
Interruptive alert that triggers with a diagnosis of acute bacterial sinusitis and when a nonoptimal antibiotic regimen (i.e., antibiotics that do not include amoxicillin/clavulanate or doxycycline, and/or an antibiotic duration greater than 5 days) is prescribed. The alert is prepopulated with a 5-day antibiotic duration.

The HFH ambulatory ASP developed an interruptive alert (ie, OurPractice advisory, Epic Systems, Verona, WI, USA) that was integrated into the EHR to optimize antibiotic prescribing for acute bacterial sinusitis (Figure 1). The interruptive alert is triggered when a provider signs an order for a nonoptimal antibiotic regimen, including non-amoxicillin/clavulanate or doxycycline antibiotic selection and/or duration greater than 5 days, with the associated ICD-10 code for acute bacterial sinusitis (J01.XX). The interruptive alert states “Amoxicillin/clavulanate or doxycycline (penicillin allergy) for 5 days is best practice for bacterial sinusitis” and displays the preferred amoxicillin/clavulanate and doxycycline (β-lactam allergy) prescriptions, prepopulated with a 5-day treatment duration. The alert maintains prescriber autonomy, who can select that an “alternative antibiotic is required” and proceed with the original antibiotic prescription. The interruptive acute bacterial sinusitis alert received support from health system and ambulatory antimicrobial stewardship committees and was implemented on April 3, 2025.

### Key definitions and data

The primary outcome was receipt of optimal sinusitis antibiotic therapy, defined as amoxicillin/clavulanate or doxycycline (β-lactam allergy) for a 5-day duration, as recommended by institutional guidelines for sinusitis treatment. Secondary outcomes included median (IQR) duration of sinusitis antibiotic therapy, in days of therapy, and median (IQR) duration taken by the prescriber to acknowledge the interruptive alert, in seconds. A nonequivalent dependent variable of sinusitis supportive care therapies was used to evaluate the standard of care over study periods, defined as documentation or prescription of nasal saline spray, antihistamines, or nasal corticosteroids.

### Data collection and statistical analysis

Patient data were acquired for screening using Epic’s SlicerDicer tool (Epic Systems Corporation, Verona, WI, USA) based on the diagnosis of acute bacterial sinusitis ICD-10 codes, an antibiotic prescription generated during the outpatient encounter, and the silenced (preintervention group) or active (postintervention group) interruptive alert. This study was designed to detect a difference in optimal antibiotic prescribing for acute bacterial sinusitis. A sample size of 200 patients was calculated using a 2-sided α of .05, β of .8, and relative anticipated effect size of 15% based on previous literature evaluating outpatient interruptive alerts, assuming a baseline prevalence of 10% optimal antibiotic prescribing.^5^

Descriptive statistics (proportion [%], median [IQR]) were used to describe patients in the pre and postintervention groups. Bivariate analyses were used to compare groups; continuous data were analyzed using Mann–Whitney U test and categorical data were compared using the Pearson X^2^ or Fisher’s exact tests. For all analyses, *P* values < .05 were considered statistically significant. All statistical tests were performed using SPSS Statistics, version 29 (IBM Corp., Armonk, NY, USA).

## Results

The silenced interruptive alert fired a total of 3,137 times in the preintervention period, and the interruptive alert fired 3,037 times in the postintervention period. The primary outcome, the prescribing of optimal antibiotic therapy, occurred in 7% of patients in the preintervention group and 30% in the postintervention group (unadjOR, 5.69; 95% CI, 2.36 – 13.72; *P* < .001). A nonequivalent dependent variable of sinusitis supportive care was observed in 66% patients in the preintervention group and 71% in the postintervention group (*P* = .447). The median (IQR) duration of therapy in the preintervention group was 7 (5 – 10) days and 7 (5 – 7) days in the postintervention group (*P* = .004). The median (IQR) time to acknowledge the interruptive alert was 7 (4 – 15) seconds. The alert was bypassed 58 times in the postintervention period; 47 instances resulted in nonoptimal antibiotic prescribing that primarily represented suboptimal duration. There was observed reduction in interruptive alert activations over the postintervention time frame.

## Discussion/Conclusion

Implementation of an interruptive alert through a tailored prescription strategy increased the proportion of optimal antibiotic therapy by 23% in ambulatory encounters for acute bacterial sinusitis. In addition, the median (IQR) duration of therapy significantly decreased, aligning more closely with institutional guideline recommendations. These improvements demonstrate that enhanced EHR tools can positively impact outpatient antibiotic use where most prescribing occurs.

While the present study demonstrated a significant increase in optimal antibiotic use for acute sinusitis, the fact remains that most acute sinusitis diagnoses are misdiagnosed and do not require antibiotic treatment.^3,6^ This study did not evaluate if antibiotics were indicated due to prolonged or worsening symptoms, however, the interruptive alert was designed to mitigate antibiotic-related harms by encouraging selection of both the optimal drug and duration. The diagnosis of acute sinusitis is challenging, as ambulatory providers often rely on nonspecific symptoms alone to guide antibiotic treatment without radiographic imaging.^6^ Patients with suspected recurrent or chronic sinusitis should be evaluated by an otolaryngologist, who will perform nasal endoscopy to assess for sinusitis or other sinonasal condition. Of note, one regional study found that half of patients referred for evaluation did not ultimately meet criteria for sinusitis.^3^

Study limitations also warrant discussion. Alert fatigue may occur as interruptive EHR alerts may be disregarded when encountered too frequently and could have limited alert impact.^7^ Additionally, this study did not account for any potential confounders that may influence prescribing practices, including patient specific characteristics or seasonality. The observed reduction in interruptive alert activations over time may reflect seasonal declines in sinusitis visits or improved prescribing practices, with fewer prescriptions requiring intervention.

The study findings support the effectiveness of leveraging EHR tools to improve sinusitis care, and specifically to optimize antibiotic prescriptions. Future studies should further enhance EHR available features in sinusitis diagnoses, including an interruptive alert for recurrent acute and chronic sinusitis to promote early referrals to otolaryngologists for evaluation. Additionally, given the brief time required for interruptive alert acknowledgement, future evaluations should assess the potential time savings for providers achieved through streamlined adherence to evidence-based care and integrated antibiotic prescribing workflows. This study supports the use of interruptive alerts to provide real-time decision support across outpatient settings and represents a scalable strategy for health systems to optimize antibiotic use and advance stewardship.
